# Stakeholders’ views on the strengths and weaknesses of maternal care financing and its reform in Georgia

**DOI:** 10.1186/s12913-017-2485-8

**Published:** 2017-08-08

**Authors:** Lela Shengelia, Milena Pavlova, Wim Groot

**Affiliations:** 1 Department of Health Services Research, CAPHRI, Maastricht University Medical Center Faculty of Health, Medicine and Life Sciences, Maastricht University, Maastricht, The Netherlands; 2 The National Center for Disease Control and Public Health of Georgia, Tbilisi, Georgia; 3 Georgian Association of Gynecologists and Obstetrics, Tbilisi, Georgia; 4 Top Institute Evidence-Based Education Research (TIER), Maastricht University, Maastricht, The Netherlands; 5 Varketili 3, IV 401 aprt.75, Tbilisi, Georgia

**Keywords:** Maternal health, Health system, Reform, Financing, Out -of –pocket payment, Georgia

## Abstract

**Background:**

The improvement of maternal health has been one of the aims of the health financing reforms in Georgia. Public-private relationships are the most notable part of the reform. This study aimed to assess the strengths and weakness of the maternal care financing in Georgia in terms of adequacy and effects.

**Methods:**

A qualitative design was used to explore the opinions of key stakeholders about the adequacy of maternal care financing and financial protection of pregnant women in Georgia. Women who had used maternal care during the past 4 years along with health care providers, policy makers, and representatives of international partner organizations and national professional body were the respondents in this study. Six focus group discussions to collect data from women and 15 face-to-face in-depth interviews to collect data from the other stakeholders were conducted. Each focus group discussion consisted of 7–8 women. Two focus group discussions were carried out at each of the target settings (i.e. Tbilisi, Imereti and Adjara). Women were selected in each location through the hospital registry and snowballing method.

**Results:**

The evidence shows that there is a consensus among maternal care stakeholder groups on the influence of the healthcare financing reforms on maternal health. Specifically, the privatization of the maternal care services has had positive effects because it significantly improved the environment and technical capacity of the maternity houses. Also, in contrast to other former-soviet republics, there are no informal payments anymore for maternal care in Georgia. However the privatization, which was done without strict regulation, negatively influenced the reform process and provided the possibility to private providers to manipulate the formal user fees in maternal care. Stakeholders also indicated that the UHC programs implemented at the last stage of the healthcare financing reform as well as other state maternal health programs protect women from catastrophic health care expenditure.

**Conclusion:**

The results suggest a consensus among stakeholders on the influence of the healthcare financing reform on maternal healthcare. The total privatization of the maternal care services has had positive effects because it significantly improved the environment and the technical capacity of the maternity house. However, the aim to improve maternal health and to reduce maternal mortality was not fully achieved. Financial protection of mothers should be further studied to identify vulnerable groups who should be targeted in future programs.

**Electronic supplementary material:**

The online version of this article (doi:10.1186/s12913-017-2485-8) contains supplementary material, which is available to authorized users.

## Background

Many countries could not reach the Millennium Development Goal (MDG) for Maternal Mortality Ratio (MMR) although remarkable improvements have been observed [1]. Financial accessibility to appropriate maternal care has been identified as one of the important determinants of the state of maternal morbidity and mortality [2]. Like in the MDGs, the equitable access to maternal care has also been given ample importance in the new Sustainable Development Goals (SDGs) because of its social, health and economic development impacts on households as well as countries’ health system [3].

The target for MMR (i.e. 12 /100,000 live births) has not been achieved and it amounted to 36/ 100,000 live births in 2015 [3]. Since the independence in 1991, like other former Soviet countries, Georgia has gone through several phases of health financing reform to improve access to health care, including maternal care. These reforms have influenced the utilization of health care services due to an increase in the burden of formal fees for services applied in the context of informal patient payments [4, 5]. Total privatization of the health system, including purchaser-provider split, is the most noticeable outcome of the reforms [6].

Privatization of the health system resulted in the transformation of the traditional centralized Semashko model. However, it was implemented in a weak state capacity to regulate the private market. Private providers’ interest in making profits ‘potentially compromise patients’ health and economic safety. Therefore, the government is investing a considerable amount of money to purchase health care including maternal care from private providers. This is also in line with the current reform for the implementation of the Universal Health Care (UHC) program [7]. Nearly 7.4% of GDP in 2014 is allocated to health care [8].

The Ministry of Labour, Health and Social affiars (MoLHSA) is the state agency, which receives the general government health budget to purchase health care for the population, including maternal care, from the private providers. Maternal care in Georgia is provided by a countrywide network of women consultation centers (WCC) and maternity houses. WCCs are primary level facilities that provide only antenatal care. The maternity houses are secondary level facilities providing antenatal care, physiological childbirths, Caesarian sections (C-section) and emergency obstetric care.

The MoLHSA allocates a certain part of total Government Health budget to implement maternal care through multiple agencies which are responsible for different vertical and horizontal maternal care programs. For example, the National Center for Disease Control and Public Health of Georgia (NCDC) purchases the logistics for antenatal screening tests and distributes money to private providers. Along with the services, as mentioned above, the following other free-of-charge maternal care services are also included: antenatal screening for HIV, Hepatitis B and C, and syphilis, folic acid and iron supplementations, physiological childbirths and C-sections [9]. The social agency is responsible for the provision of antenatal care, early detection and management of high risk pregnancy and congenital anomalies. The same agency is responsible for payments for both physiological childbirths and C-sections as part of the UHC program. Practically, the provision as well as financing of maternal health programs is fragmented because of the involvement of several agencies controlling vertical and horizontal maternal care programs.

Despite publicly provided four free-of-charge antenatal visits as well as childbirth services, out-of-pocket payments (OPPs) remained a considerable burden for households because of additional antenatal care visits and fees for “personal doctors”. For example, the average OPPs for a C-section and a physiological childbirth were 667.2 GEL and 385.3 GEL respectively [10]. Women in the highest income quartile paid higher OPPs for childbirth of any type than the lowest quartile [10, 11]. Moreover, the cost of medicines is nearly three times higher than the average cost in other European countries which is also directly linked to high OPPs [12]. According to Curatio International Foundation, the high OPPs are related to delays in medications and the utilization health care services such as outpatient care, hospital and additional maternal care services, which are not included in the UHC program [11].

The goal of the health financial reform is equitable access to health care; thereby, health and economic protection for the population. In Georgia, nearly 20.1% of the total population lives below poverty level [13]. The present OPPs in maternal care in the private market present a risk, which may hinder the health and socio-economic protection of households and may also be an obstacle for achieving the goals of the state UHC program. An in-depth investigation is essential to assess the key stakeholders’ opinions about strengths and weakness of the current maternal care financing. Thus, this study aimed to assess the strengths and weakness of the maternal care financing reforms in Georgia in terms of adequacy and effects.

## Methods

We used a qualitative design to explore the opinion of key stakeholders about the adequacy of maternal care financing and financial protection of pregnant women in Georgia. The study was conducted in May and June 2015 in the capital Tbilisi and in two regions of Georgia, namely Imereti and Adjara. Women who had used maternal care during the past 4 years along with health care providers, policy makers, and representatives of international partner organizations and national professional body were the respondents in this study. An ethical approval of the study was obtained from the National Center for Disease and Public Health of Georgia.

We conducted six focus group discussions (FGDs) to collect data from the target women, and 15 face-to-face in-depth interviews to collect data from the other stakeholders. Each FGD consisted of 7–8 women. Two FGDs were carried out at each of the target settings (i.e. Tbilisi, Imereti and Adjara). The target women at each location were divided into two groups; one group with women had one child, and another group with women had more than one child. This was done for a better understanding of the differences in the women’s experience of childbirth/s based on parity (i.e. primipara or multipara).

In each region, two research assistants identified the target women. We selected the target women in each location through the hospital registry and snowballing method. Two-thirds women were sampled from the selected hospital registries consecutively provided they fulfilled the inclusion criteria. Random sampling was not possible since there were no separate registries only for the target mothers, thus no usable sampling frames were available. Information from those mothers was used to identify the target mothers in the community which allowed sampling the rest one-third mothers through snowballing. We did this to enhance the possibility of selecting women who gave childbirth at different facilities rather than a single facility; thereby, to get data from women with diverse experience of maternal care. There were no age or economic status stratification criteria applied to allow exploring the opinion of women in reproductive age representing diverse socio-economic groups. For in-depth interviews with the other stakeholders, we selected three policy makers, three private health facility representatives, two representatives of international organizations partnering in the development of maternal care, one representative of national professional organization and six maternal care providers (physicians). We used convenience sampling method to select target mothers and purposive sampling method respondents of in-depth interview.

The objective was to investigate the adequacy of maternal care financing and economic protection of pregnant women from the perspective of different stakeholders. Focus group and in-depth interview discussion topics were formulated using primary literature review. The guides were developed in English (please see Additional files 1 and 2) and then translated into local Georgian language by the principal investigator. The data collection instruments (i.e. the guides) were pre-tested. FGD and in-depth interview guides were pre-tested and adapted as needed.

Informed written consents were given by all respondents prior to each FGD and interview. All FGD sessions were conducted by a Facilitator with longstanding experience of conducting FGDs. The Principal Investigator acted as moderator for the FGDs and, conducted all in-depth interviews. Confidentiality of the collected data was maintained. Each session was audio-tapped. The need of audio taping of each FGD session and in-depth interview was explained to all respondents and permission for recording was obtained. All FGDs and in-depth interviews were conducted in Georgian language. Which were translated into English by an English language expert. The Principal Investigator compared and validated the Georgian and English versions of the transcripts. Data was collected during May and June, 2015.

We investigated stakeholders’ opinion about strengths and weakness of the current maternal care financing reform in terms of its adequacy and effects. We applied the method of directed qualitative content analysis as defined by Hsieh and Shannon [14]. Specifically, the focus was on identifying data in the transcripts related to two main themes: [1] arguments in support of the current maternal care financing, and [2] arguments against the current maternal care financing. We extracted and analyzed the data manually. An abstract coding approach was applied to develop a set of codes [15] related to the two themes. Two researchers independently coded the data based on the key attributes of financing of maternal care as outlined above and consensus on any discrepancies were built through discussions. The results were synthesized in a narrative manner.

## Results

A total of 41 women (primipara *n* = 19; multipara *n* = 22) participated in six FGDs and 15 other stakeholders (i.e. policymakers; *n* = 3; health insurers, *n* = 2,;providers,*n* = 4; and representatives of national professional, *n* = 1; and international organizations, *n* = 2; and physicians, *n* = 3) participated in in-depth interviews. Below, we present the key stakeholders’ opinions about strengths and weaknesses in terms of adequacy and effects of the current maternal care financing reform.

### Arguments in support of the current maternal care financing

- Adequacy in terms of financial allocation and maternal care service package:

Policy makers, providers and physicians mentioned that the implementation of the UHC program as part of the current health financing reform, results in an improved financial allocation in health care. Also majority of the study participants stated that the financial protection of the population in health care including maternal care has been improved. According to policy makers, through the UHC program and several vertical programs, the state has improved coverage for maternal care as well as financial protection of households. The target mothers also supported this statement; additionally, some mothers stated that the current UHC program met most of their needs related to childbirth. Relevant quotes are presented in Table 1.

**Table 1:** Statements in support of the current maternal care financing reform

**In- depth interviews**
The fees should apply for additional services such as food and room or patient comfort [1]
The state covers everyone pretty much; the high risk pregnancies are covered by vertical program [1, 2, 6]
I am for co-payment. I think 10-20% of co-payment could be existed. Co-payment also means some kind of responsibility from the client’s side. But it should not be burden for the population [3]
Privatization supported to the legalization of incomes [5]. Informal payments have been eliminated [6].
Some people cannot pay. Therefore, State should provide full coverage of maternal care [15]
**Focus group discussions**
Families are trying to be prepared for the childbirth and most of the families are ready for payment [16, 17]
I had private insurance. It helped me to pay everything, except pharmaceuticals [16]
We gave to the doctor a gift as a token of gratitude [16, 17].
I had complicated childbirth and was transferred to Tbilisi. The total cost was covered by the State completely [21].

Policy makers mentioned that geographical and financial access to maternal care has improved. They stated that basic antenatal care (i.e. four visits), high risk pregnancies and transportation in case of pregnancy complications are covered under the State vertical maternal care program and UHC covers childbirths (i.e., physiological childbirth and C-section). Respondents of in-depth interviews also indicated that any additional services which are not included in the vertical and UHC programs: for example, antenatal care more than four visits, preeclampsia and near miss services need to be paid by OPPs. All respondents mentioned that pregnant women have the freedom of choosing facilities and providers, which are not included in the State programs for example: personal doctors, and medical investigations. However, they have to pay additional amounts beyond state allocation.

According to most of the respondents, the private health insurance supplements the maternal care program; however the predominant opinion was that all individuals who are able to pay have to purchase insurance. This will facilitate the State to expand the package for the poor and the unemployed people (Table 1).

- Maternal care financing versus out-pocket-payments:

Participants of in-depth interviews stated that despite the UHC program and vertical programs, pregnant women are commonly paying for additional services by themselves. OPPs are mostly related to medications and diagnostics, which are not included in any of the State programs. According to the women, the presence of OPPs was preferable if the pregnant women were asking for additional services, such as private room, personal doctor etc. Some providers think that OPPs prevent overuse of services. Women mentioned that family and relatives are the main sources of OPPs. One woman said – “pregnancy is expected and improves status of women in families. For this, families and relatives are willing to pay any extra costs relating to maternal care”.

Informal payment in health care was a major financial burden for households previously, which is now completely eliminated due to new laws and the influence of the privatization in health care. However, sometimes mothers and families present gifts to doctors/nurses as a part of gratitude, which is not an informal payment; instead it is an expression of good patient-doctor/nurse relationship.

### Arguments against the current maternal care financing

- Financial allocation versus maternal care package:

According to some private providers, the current allocation of 55 Gel (equivalent to 20 Euro) for four antenatal visits is not sufficient to provide good quality of care. Also often four antenatal visits and the content of services did not meet the needs of all pregnant women. Because of this limited allocation and service contents, early detection of high risk pregnancies are often missed. In support of this statement, one woman mentioned that “I needed extra antenatal visits and tests and all costs were provided by my family”. One of the privet providers mentioned that the government allocation for specific services is quite marginal to make profit. This often compromised private providers’ interest of joining public health care programs.

Some policy makers and providers expressed concerns about the quality of maternal care especially antenatal care, due to the current financing reform. Relevant quotes from the transcripts are presented in Table 2. Providers also mentioned that the current financing system has fragmented the State maternal care programs. For example, one agency is purchasing antenatal screening tests for HIV, Hepatitis B and C, syphilis, while another agency is responsible for the implementation of the antenatal checkup.

**Table 2:** Statements against the current maternal care financing reform

**In-depth interviews**
I think all people who work have to purchase insurance package and plan pregnancy. But if person does not have income the maternal care should finance by the government [3].
She needs to do screening on bacteriuria but she said “I don’t have money” and the doctor reported that “she refused screening” [10].
Near miss services should not have any OPPs because this is something you cannot predict or plan [4]
**Focus group discussions**
I was asked for additional lab tests. Lastly I found that was not necessary. I complained to MoLHSA and the facility was penalized [17]
Private providers are increasing fees frequently and suggesting more consultations than needed [20].
Only emergency services are financed but not preventive measures even for high risk pregnancies [17]

Some of the maternal care providers as well as policy-makers indicated that the government should finance only the poor population by providing them with a wider package and others should pay OPPs for maternal care services. In the current situation, the equal financing of poor and rich people lead to the problem of regressive financing. Representatives of national and international organizations questioned the regulation of financing of C-section. “The number of C-section has significantly increased in the country” according to one of the key stakeholders. Many C-sections are conducted because of either demand of women or providers’ income interests rather than real indication. Use of state resources for such unjustified C-sections causes waste of resources. The government should strictly regulate this issue for the proper use of resources.

- Opinions about OPPs:

Two women mentioned that specialized laboratory tests are not included in the current government programs and some are not even available in the country. One of them mentioned-“I was suggested for a genetic test which was expensive and not available in Georgia. The doctor asked me to send sample to Germany. It was costly and I could not manage to do that.” Some providers mentioned that the burden of OPPs is significant for maternal care in case of complications. The State program for the management of pregnancy complications exists but the program has very strict inclusion criteria. According to the opinion of one of the providers, management of near-miss cases is often difficult with the available facilities in the country. However, most of the cases might be prevented if necessary measures are taken on-time. Moreover, there is no rehabilitation program for women who undergo near miss cases. During the FGDs in Kutaisi and Batumi, women actively raised the issue of pregnancy-related complications. Women from rural areas mentioned that some of them faced various types of complications such as bleeding and preeclampsia, and they were transferred to tertiary level facilities in region or in Tbilisi. According to them, due to severe complications, they did not pay and the government covered all expenses.

- Regulation:

A few women expressed their dissatisfaction because of the strict rule of registering before 12 weeks of pregnancy. One of the mothers said that women may not be registered with the system because of different reasons and for this they should not be deprived from state provision of financial support. She mentioned- “I was not given a voucher because of attending the clinic at 13 weeks of pregnancy. I did not do it intentionally but I could not manage to go before 12 weeks because of family problems”.

Strengthening the regulation in the healthcare sector and particularly maternal care is essential according to the participants. One of the private providers mentioned that because of the cost-of transportation, pregnant women delay referrals and this negatively affects the outcome of maternal care as well. The transportation system is not included in the UHC program.

## Discussion

This paper describes stakeholders’ opinions about the strengths and weakness in terms of adequacy and effects of maternal care financing reforms in Georgia. The study gives us an opportunity to examine the influence of the privatization on maternal health in Georgia. All stakeholders indicated that the recent financial reform in the health care sector has decreased the financial hardship for mothers. But also, there is consensus among the groups that in case of pregnancy complications, and personal choice of facility and provider out of state programs, the burden of the OPPs is significant.

Privatization plays a crucial role in service provision in the health care sector. In Georgia, it influences the development of the health care system. As in other European countries, the privatization is a response to public sector failure [16]. The decision of the government to privatize the entire health sector is an outcome of a policy-driven process, but it is not followed by strong regulation mechanisms and this gives room to private ownership of health facilities, particularly owners of the maternity houses, to manipulate the user fees. Specifically, our study shows that there is consensus among stakeholders that the latest decision of the government to implement UHC program protects mothers from financial burden. However, the weaknesses in regulation are also observed.

Specifically, the privatization of the healthcare service, particularly in the maternal care field, has improved the infrastructure. Some authors argue that privatization in the healthcare sector simulates competition, which leads to the improvement of quality of care and the service package [16]. In Georgia, competition between the private maternity houses is mainly associated with improvements in technical efficiency and the infrastructural capacity of the facilities. Opposite to Georgia, Armenia and Ukraine maintained a public health system similar to that established during the Soviet era with a focus on curative care. In spite of the fact that post-soviet countries chose their own path of developing their own health system, all countries experience the same problems and challenges in maternity care.

As our study shows, one of the advantages of the privatization in Georgia is the abolishment of the informal payments. In the country, informal payments existed during the soviet era and became more common in the early 1990’s. Even at the beginning of the 21 century, informal payments were directly paid to the provider, were demanded by providers as well as influenced by a soviet culture of gratitude [17]. Informal payments provided a way to obtain medical care immediately [18]. This is also confirmed by our findings. Thus, the elimination of informal patient payments is not the result of a more efficient resource allocation or more adequate regulation by the State, but an outcome of private owners’ efficient managerial capability in this regard. At the same time, there is evidence that in other post-soviet countries such as Ukraine, informal payments for maternal care are still widely spread [19].

Nevertheless, our findings indicate that formal OPPs are a significant burden for pregnant women in Georgia. During the focus group discussions, some mothers mentioned that beside the initial payments, they were requested to pay some additional amounts for additional services in case of complications, which they paid officially. Moreover, respondents of the in-depth interviews also stated that during the antenatal period, most women required additional visits and because of this, they paid extra out of pocket. The OPPs are also increasing due to phenomenon of “personal doctor”. The phenomenon is not unique for Georgia.

Women have autonomy of choosing facility, provider and diagnostics, and even mode of delivery location, then they are also kept responsible for the payment, when they have the ability to pay. Thus, the current financial reform is regressive since both the poor and the rich are getting equal state facility. Whenever needed, the richer segment has access to specialized services through OPPs but the poor segment is not supported by state program for the specialized services. A progressive financing system could protect both the poor and the rich pregnant women.

Women in Ukraine and Armenia also use this type of service but in these countries, they mostly pay for it informally [19, 20]. We identify in our study that the main push factors to search and pay extra for a personal obstetrician are: safety, responsiveness and personal comfort. It is clear from our study that pregnant women and their families prefer to pay more for the service of a personal obstetrician and ensure “high quality of care”. However, they choose a personal obstetrician according to their ability to pay as well as taking into account direct and indirect costs. This situation leads to inequities and disparities among mothers as not everybody can afford a personal physician and might receive substandard care. For example, one of the participants of the focus group discussion mentioned that her mother had to pay all expenses for the last pregnancy. Moreover, epidemiological surveillance of maternal mortality done by the NCDC of Georgia showed that maternal mortality is higher among lower middle and low income groups because lower income groups utilize fewer maternal care services due to direct and indirect cost [21]. This is found in our study as well.

The implementation of the UHC program in Georgia is influenced by an increased burden of OPPs and decreased utilization of health services [6, 22]. UHC, including the coverage of maternal care, remains a priority in the post-2015 agenda [23]. The goal of the UHC implementation in Georgia is to protect the health of the entire population and to promote a sustainable economic and social development, as it is targeted by WHO in 2010 [24].

Our study shows that after the implementation of the UHC program, the utilization of healthcare services became easier and catastrophic health care expenditure reduced as the UHC program finances childbirth and C-section services. In Georgia, the share of C-sections is high (41.5% in 2015) compared with European countries [25]. According to participants in the focus group discussions and in-depth interviews, the State finances C-section on demand. The UHC program pays 500 GEL for a C-section performed on demand of the patient and 800 GEL in case of a medical indication. However, the price for a C-section on demand is the same as for a normal vaginal delivery. However, the potential short-term maternal outcome of a vaginal delivery compared with elective C-section includes a shorter length of hospital stay, lower infection rates, fewer anesthetic complications, and higher breastfeeding initiation rates [26]. Beside this, our study shows that the fees for services of childbirth and C-section varied from 900 to 3500 GEL among healthcare providers. The variation in the fee-for-service rates depends on how famed the maternity house is and what additional comfort they provide to the users. However, the UHC financing of any type of childbirth protects the mothers and their families from catastrophic health expenditure. Compared to Georgia, C-sections are lower in Ukraine and Armenia; two former-soviet states. In 2013, the number of C-sections per 1000 live birth was quite high and reached 371.09 in Georgia while it was 238.07 and 168.88 in Armenia and Ukraine respectively [27].

The opponents of the positive influence of maternal care financing on maternal health are quite open about the negative effect of the fragmentation of the vertical and horizontal maternal health programs due to high administrative costs. This raises the issue of efficiency [28]. In this situation, the organization of antenatal care through several agencies without a monitoring and evaluation mechanism needs attention from the policy makers. Since 1997, Georgia offers 4 free antenatal services in accordance with WHO recommendations [28, 29]. Many maternity houses do not participate in the program because of two reasons: first, because of insufficient compensation per package and second, the service content of the antenatal care package does not cover all antenatal care needs. Mothers as well as providers and representatives of national and international organizations, strongly advocate an increase of the antenatal care package financing. Since years, the antenatal package has remained the same and the government pays the same amount (55 GEL) [7] in spite of the inflation and changes in user fees. Almost all women-participants mentioned that they visited antenatal care clinics at the request of the physician nearly 10 or more times, and paid OPPs. This finding indicates that the interest of the maternity houses that participate in the State antenatal care programs is to recruit pregnant women and then encourage them to utilize more services than necessary. All these findings indicate that there is supply-induced demand in Georgian maternal care and a providers’ interest to increase their income. This raises the question of efficiency and effectiveness of the maternal healthcare programs. The fragmentation of maternal healthcare programs and high pharmaceutical costs are common in Ukraine and Armenia as well. Both countries are facing challenges in the equity of healthcare financing [14, 30].

Universal coverage of maternal care reflects the individual rights of pregnant women and social solidarity [31]. However, it should focus on equity and should take into account the social determinant and needs of subgroups and those who are vulnerable [3]. Georgia is a lower middle income country. In 2014, GNI per capita was 4490.00 [31]. In this situation, the burden of UHC without the regulation and monitoring mechanism is significant for the country.

### Strengths and weakness of the study

We triangulated the stakeholders’ opinions to strengthen the validity and reliability of the results. A wide range of stakeholders were included in the study that gives a real picture of maternal care in Georgia. However, a small number of settings were included in this study. Thus, the results cannot be extrapolated to the entire country. However, as in any qualitative study, the primary research objective is to collect in-depth information on the views of the stakeholders rather than achieving representatives for the country. Accordingly, the results are important because they provide an in-depth understanding of the problem. Further, experienced facilitators managed the FGDs and also explored the in-depth opinions of the target women. Thus, we mitigate the facilitator-related bias by choosing an experienced moderator and interviewers, and by applying a guide to assist them during the data collection process. Also, we pre-tested the guide before the field works. By involving experts in the translation process, we diminish such bias to a certain extent.

## Conclusion

This qualitative study was done in two regions and in Tbilisi and aimed to elicit stakeholders’ opinions about maternal health financing in Georgia. The results of our study suggest a consensus among stakeholder groups on the influence of the healthcare financing reform on maternal healthcare. The total privatization of the maternal care services has had positive effects because it significantly improved the environment and the technical capacity of the maternity house. But the privatization was done without strict regulation, which negatively influenced the reform process and provided the possibility to private providers to manipulate user fees in maternal care.

Stakeholders also indicate that the UHC program implemented at the last stage of the healthcare financing reform protects the mothers from catastrophic health expenditure. Besides UHC, the State implemented several vertical maternal health programs and maintained financial access to basic maternal healthcare services. These program protect pregnant women from catastrophic health care spending for maternity care. In addition, stakeholders reported that the healthcare reforms eliminated the informal payments. However, vulnerable groups are facing difficulties in paying the formal fees for some lab tests that are not in the basic package and also because of transportation cost for antenatal care. As study participants indicated, an increase in the basic antenatal care package and its financing, as well as strengthening the regulation in the healthcare sector, especially regarding the unjustified use of C-sections, are essential for Georgian maternal healthcare services.

In spite of the significant steps taken by the government to improve maternal health and to reduce maternal mortality, the target was not achieved. Therefore, the financial protection of mother should be further studied to identify the needs of the vulnerable groups who should be targeted in future programs.

## Additional files

Additional file 1: Guide for In-depth Interview. The Guide for In-depth Interviews was used to study stakeholders’ views on the strengths and weaknesses of maternal care financing and its reforms in Georgia. (DOC 88 kb)

Additional file 2: Guide for Focus group discussions. The Guide for Focus group discussions was used to study stakeholders’ views on the strengths and weaknesses of maternal care financing and its reform in Georgia. (DOC 100 kb)

## Abbreviations

C-section: Caesarian sections
GDP: Gross domestic product
HIV: Human immunodeficiency virus
MDG: Millennium Development Goal
MMR: Maternal Mortality Ratio
MoLHSA: Ministry of Labor, Health and Social Affairs
NCDC: National Center for Disease and Public Health of Georgia
OPP: Out-of-pocket payments
SDGs: Sustainable Development Goals
UHC: Universal health coverage
WCC: Women Consultation Centers
