# Supplementary material for: Stakeholders’ views on the strengths and weaknesses of maternal care financing and its reform in Georgia
Source: BMC Health Serv Res. 2017 Aug 8;17:544. doi: 10.1186/s12913-017-2485-8 (PMC5549305; doi:10.1186/s12913-017-2485-8)
Supplement: Supplementary file 1 — Guide for In-depth Interview. The Guide for In-depth Interviews was used to study stakeholders’ views on the strengths and weaknesses of maternal care financing and its reforms in Georgia. (DOC 88 kb) [file 12913_2017_2485_MOESM1_ESM.doc]

**The Guide for In-depth Interviews was used to study stakeholders' views on the strengths and weaknesses of maternal care financing and its reforms in Georgia**

**GUIDE FOR IN-DEPTH INTERVIEWS**

Below is a general guide for leading the in-depth interviews. This guide may be slightly modified as needed (e.g. for the following interview based on the previous one) but without changing the content of the questions. During the interview additional clarifying questions can be posed. Before the interview, note down the code of the interview.

## **INTRODUCTION (5 MIN)**

- **Thank the respondent and introduce yourself.**

*Thank you for accepting our invitation for this interview. Your participation is highly important to us. Let me first present my self: … The interview should last about 60-70 minutes.*

- **Explain the general purpose of the interview.**

*The questions prepared for this interview concern your opinion and attitudes related to the maternal health. This interview and similar interviews with policy-makers, international and national NGOs, health insurance representatives and service providers are carried out for a research project focused on the assessment of maternal health in Georgia. Based on the results of these interviews will be made with purely scientific objectives.*

- **Explain the process of the interview.**

*There will be several main questions during the interview.*

*This interview has purely academic purposes. Information discussed is going to be analysed as a whole and respondent’s name will be never used in any analysis of the discussion or in any report.*

- **Explain the presence and purpose of recording equipment and introduce observers.**

*I will need to tape record the discussion because I would not want to miss any of your comments. No one outside of this room will have access to these tapes.*

- **Ask the respondent to sign the Informed Consent form.**

*All details that I have just explained are described in our Informed Consent form. Please, read this form and sign it to confirm that you agree to participate.*

Give the respondent 2 copies of the Informed Consent form filled in and signed by the principle investigator. Collect one of the forms signed by the respondent.

- **Make sure that the respondent is ready to start.**

*Do you have any questions? May we turn on the tape recorder?*

Turn on tape recorder and pronounce the code of the in-depth interview.

**II. INITIAL DISCUSSION ON maternal healthcare in Georgia (20 MINUTES)**

- **Describe again the purpose of the interview.**

*Let us get started. As I have already mentioned, this interview is focused on maternal healthcare in Georgia. We are specifically interested in your own perspective on this topic.*

- Clarify the definition of maternal healthcare.

*In this interview, we will consider maternal health care services. Maternal health refers to the health of women during pregnancy, childbirth and the postpartum period.*

- *Describe the State health care programs that cover maternal healthcare services in Georgia*

*There are several maternal health care vertical programs:*

*Maternal and child health program that covers:*

- *Four antenatal visits*
- *Screening of all pregnant women on HIV, Hepatitis B and Gonorea and conformation of positive cases*
- *Early detection and examination of high risk pregnant women on genetic disorders by screening with triple test* at 14-16 weeks of pregnancy,
- *Pre-, intra and post-partum health care of high risk pregnant women*
- *Management of pregnancy and delivery complications during first six days*
- *Treatment of post-partum syphilis*
- *Invasion examination of pregnant women -Amniocentesis karyotype method*
- *“Health for All” ensure free delivery and C-section*

- Discuss the attitudes of the respondent towards the State maternal health care program
- *Do you think that the State maternal health care program covers all needs of pregnant women and young mothers? Why?*
- *What about health insurance, which part of maternal healthcare does it covers? Is it sufficient or not?*

After the responses from this prompt have been exhausted, move on.

- Discuss the attitudes of the respondent towards out of pocket payments in maternal health care services.
- *co-payments*
- *user fees,*
- *user charge*
- *fee for services*
- *informal payment*
- *payment for pharmaceuticals*

Give examples of official patient payments in antenatal clinics or in maternity houses

After the responses from this prompt have been exhausted, move on.

- Discuss the attitudes of the respondent towards the burden of out of pocket payment and co-payment in maternal care services.

*Do you think that out of pocket payment should exist in Georgia, Why?*

*Do you think that co-payment should exist in Georgia, Why?*

- Discuss the attitudes of the respondent towards payments for given services.

*Do you think that payments should be applied to the following services?*

Show Card 1 to the respondent.

| CARD 1 | - - 1. ante natal care     2. service of specialist     3. delivery services     4. near miss services     5. post natal care | 2015 Georgia |
| --- | --- | --- |

If not, why not?

If it happened do you think that pregnant women will be able to pay for these services?

After the responses from this prompt have been exhausted, move on.

- Elicit opinion about the level of payment fees for maternal care

- If payments for maternal care exist in your country:

Show Card 1A to the respondent and present the levels of maternal care payment in your country for ante natal care, service of specialist, delivery services, near miss services and post natal care.

Card 1A should be provided by the project members.

| CARD 1A |  |  |  |  | 2015 Georgia |
| --- | --- | --- | --- | --- | --- |
|  |  | Services | Patient payment fee |  |  |
|  |  | ante natal care |  |  |  |
|  |  | service of specialist |  |  |  |
|  |  | delivery services |  |  |  |
|  |  | near miss services |  |  |  |
|  |  | post natal care |  |  |  |
|  |  |  |  |  |  |

*Do you think that these levels of fees are adequate for the patients?*

Show again Card 1 to the respondent and ask about the opinion of the respondent in general:

*What levels of payment would be affordable for these services for patients?*

After the responses from this prompt have been exhausted, move on.

*According to you what should be the types of payments in Georgia for* ante natal care, service of specialist, delivery services, near miss services and post natal care*? Please explain your opinion?*

- *what are other ways to extend of payment.*

Show again Card 1 to facilitate the answer.

- Discuss the role of the MoLHSA in maternal care financing and quality of care in Georgia.

Show Card 2 to the respondent and explain briefly the objectives presented there.

| CARD 2 | 1. Discouraging unnecessary use of health care services 2. Generating additional resources for the health care system 3. Allowing hospitals/clinics to generate additional resources 4. Increasing the income of individual health care providers 5. Controlling the overall health care expenditure | 2015  Georgia |
| --- | --- | --- |

*According to you, what should be the primary policy objectives to protect pregnant women and their households from health care expenditure in Georgia?*

*According to you, what should be the primary policy objectives to provide high quality maternal care services in the whole country?*

After the responses from this prompt have been exhausted, move on.

**III. GENERATION OF A LIST WITH ASSESSMENT CRITERIA (20 MINUTES)**

**• Ask the respondent to suggest relevant assessment criteria**

*Imagine that you need to assess the adequacy of financial spending on maternal care services that are implemented or considered for implementation in a country. What assessment criteria would you take into account?*

Show Card 2 to the respondent.

| CARD 3 | Imagine that you need to assess the adequacy of maternal healthcare services that are implemented or considered for implementation in a country.  What assessment criteria would you take into account?   - Safety - Delay of care - Accountability - Confidentiality - Fee for services - Consultation time - Facility outlook | 2015  Georgia |
| --- | --- | --- |

*Please, keep in mind that for us, there are no right or wrong answers. We are interested in your personal opinion. Please, also keep in mind that we aim at a broad range of assessment criteria (incl. economic, social, institutional, historical geographical, ethical, cultural, demographic and sector-specific criteria).*

- Discuss the capacity of the maternal care service providers

Discussion about the influence of manpower, referral/ transportation system and technical capacity on quality of care.

Show Card 4 to the respondent and explain briefly the objectives presented there.

| CARD 4 | 1. low workload of the facility that has less than 300 deliveries per year 2. Obstetricians, neonatologists and anesthesiologists are not available around the clock and 7 days a week in low performing facilities 3. There is lack of skilled manpower in the facilities, not every doctor can perform independently 4. Due to low salary, obstetricians, neonatologists and anesthesiologists are working in several hospitals 5. Weak referral and transport system | 2015  Georgia |
| --- | --- | --- |

*According to you, what is the main obstruct for the quality of maternal care. Which of the issues listed in the CARD 6 stated as the problem in Georgia?*

*If any, According to you, what should be done to solve the problems?*

- Discuss the outcome of maternal healthcare in Georgia

Discussion about the outcome of the maternal care

Show Card 5 to the respondent and explain briefly the objectives presented there.

| CARD 5 | 1. maternal mortality  2. Management of Pregnancy Related Hypertension   1. Infant mortality 2. Rate of C-section 3. Proportion of hemorrhage 4. Proportion of low birth weight 5. Proportion of preterm delivery 6. Proportion of women with systemic infection or in postnatal period, including readmissions   severe | 2015  Georgia |
| --- | --- | --- |

- Discuss the outcome of maternal healthcare in Georgia with European counties

*In your opinion, is there any difference among maternal health indicators of Georgia and European countries?*

*If yes, please express your opinion.*

**• Discuss the measurability of each criterion.**

Prompt discussion about the measurability of each criterion. If the respondent thinks that a given criterion is difficult to measure, ask him/her to suggest another proxy that can be measured.

After the responses from this prompt have been exhausted, move on.

- ***Discuss the attitudes of the respondent towards the privatization and marketization***

*-in your opinion, does privatization influence maternal healthcare? if yes, how?*

*- In your opinion, does libarization of the health market influence maternal healthcare? if yes, how?*

*-in your opinion, how deregulation influence maternal healthcare?*

*-in your opinion, how all these changes influence quality of the maternal healthcare?*

*- in your opinion, how all these changes influence the access to the maternal health care services*

*-in your opinion, how the implementation of universal coverage influance maternal healthcare? In which direction?*

*-in your opinion, what are areas to be improved?*

**IV. GENERAL DISCUSSION ON PAYMENTS FOR MATERNAL HEALTH CARE SERVICES (15 MINUTES)**

- Ask the respondent to reflect on the discussion.

*Is there anything we have not discussed that seems relevant to you?*

After the responses from this prompt have been exhausted, move on.

- Ask the respondent to reflect on the link between patient payment and quality of care.

*What do you think about patient payments in the maternal health care sector?*

After the responses from this prompt have been exhausted, move on.

- Make sure that the discussion has been comprehensive.

*Would you like to make any final comments?*

After the responses from this prompt have been exhausted, move on.

## **V. CLOSING PART (5 MIN)**

- **Thank the respondent**
