# Supplementary material for: Stakeholders’ views on the strengths and weaknesses of maternal care financing and its reform in Georgia
Source: BMC Health Serv Res. 2017 Aug 8;17:544. doi: 10.1186/s12913-017-2485-8 (PMC5549305; doi:10.1186/s12913-017-2485-8)
Supplement: Supplementary file 2 — Guide for Focus group discussions. The Guide for Focus group discussions was used to study stakeholders’ views on the strengths and weaknesses of maternal care financing and its reform in Georgia. (DOC 100 kb) [file 12913_2017_2485_MOESM2_ESM.doc]

**The Guide for In-depth Interviews was used to study stakeholders' views on the strengths and weaknesses of maternal care financing and its reforms in Georgia**

**GUIDE FOR FOCUS GROUP DISCUSSIONS**

Below is a general guide for leading the focus group discussions. This guide may be slightly modified as needed (e.g. for the following focus group discussion based on the previous one) but without changing the content of the questions. During the discussions additional clarifying questions can be posed. Before the focus group discussion, note down the code of the focus group.

## **INTRODUCTION (15 MIN)**

- **Welcome participants and introduce yourself.**

*Welcome to all of you and thank you for accepting our invitation to participate in this group discussion. Your participation is highly important to us.*

*The discussion will run for about 1 hour 40 minutes including one break.*

- **Explain the general purpose of the discussion.**

*This focus group discussion is organised to elicit your opinion and attitudes related to the impact of healthcare reform on maternal healthcare in Georgia. Based on the results of the focus group discussions will be made with purely scientific objectives.*

- **Explain why the participants were chosen.**

*You have been invited to participate in the focus group discussion as representatives of a specific population namely women who delivered less than four years ago.*

- **Introduce the participants to each other.**

*Let us find out some more about each other by going around the room one at a time. Please, tell us your name and the job you have. Let us start here: …*

*Please, write your name on the paper in front of you.*

- **Discuss the process of the focus group discussion.**

*Before we start, some details about the discussion process.*

*There will be several main questions that we will discuss together. In addition to this, you will be asked to fill in a short questionnaire related to the topic of the focus group discussion.*

*Please, keep in mind that for us, there are no right or wrong answers. We are interested in your personal opinion. It is highly important that everyone speaks up.*

*We encourage you to respond directly to the comments that other participants make. Still, we would like to ask you to talk one at a time.*

*If you do not understand a question, please, let us know.*

*If we seem to be stuck on a topic, we may interrupt you and move on to make sure that we cover all topics.*

- **Address the issue of confidentiality.**

*This group discussion has purely academic purposes.*

*Information discussed is going to be analysed as a whole and participants' names will be never used in any analysis of the discussion or in any report.*

*We hope you will feel free to speak openly.*

- **Explain the presence and purpose of recording equipment and introduce observers.**

*We will be tape recording the discussion because we do not want to miss any of your comments. No one outside of this room will have access to these tapes.*

- **Ask the participants to sign the Informed Consent form.**

*All details that I have just explained are described in our Informed Consent form. Please, read this form and sign it to confirm that you agree to participate.*

Give each participant 2 copies of the Informed Consent form filled in and signed by the principle investigator. Collect one of the forms signed by each participant.

- **Make sure that the participants are ready to start.**

*Does anyone have any questions? May we turn on the tape recorder?*

Turn on tape recorder and pronounce the code of the focus group discussion.

**II. INITIAL DISCUSSION ON THE STATE MATERNAL HEALTH CARE PROGRAM (30 MINUTES)**

- **Describe again the purpose of the focus group discussion.**

*Let us get started. As I have already explained, this group discussion is focused on maternal healthcare. We are specifically interested in your own perspective on this topic.*

- Clarify the definition of maternal healthcare.

*In this discussion, we will consider maternal healthcare services.*

*These are the State healthcare programs that the State implements through healthcare facilities and other healthcare providers.*

*The government of Georgia by the State health care programmes cover the following services: 4 antenatal care check-up, free delivery/labour and caesarean section, identification and testing of high risk pregnant women.*

*The private healthcare insurance also covers some maternal healthcare services, like delivery.*

*Do you have any questions with regard to our definition of maternal healthcare?*

If necessary, clarify the definition.

- Discuss the attitudes of the participants towards the maternal health in Georgia.

*Are you satisfied with the maternal healthcare services? if yes, why? if not, what was the main problem?*

*Did exist health care services met all your needs? if yes, why? if not, what was the main problem?*

After the responses from this prompt have been exhausted, move on.

- Discuss the attitudes of the respondent towards out-of-pocket payment or co-payment for maternal health services.

*Do you think that out-of-pocket payments or co-payments should be applied to the following services?*

Show Card 1 to the participants.

| CARD 1 | - - 1. ante natal care     2. service of specialist     3. delivery services     4. near miss services     5. post natal care | 2015 Georgia |
| --- | --- | --- |

If not, why not?

If it happened do you think that you will be able to pay for these services? Will these services be affordable for you?

After the responses from this prompt have been exhausted, move on.

- Elicit opinion about the level of payment for maternal healthcare

Show Card 1A to the respondent and present the levels of maternal care payment in Georgia for ante natal care, service of specialist, delivery services, near miss services and post natal care.

Card 1A should be provided by the project members.

| CARD 1A |  |  |  |  | 2015 Georgia |
| --- | --- | --- | --- | --- | --- |
|  |  | Services | Patient payment fee |  |  |
|  |  | ante natal care |  |  |  |
|  |  | service of specialist |  |  |  |
|  |  | delivery services |  |  |  |
|  |  | near miss services |  |  |  |
|  |  | post natal care |  |  |  |
|  |  |  |  |  |  |

*Do you think that these levels of fees are adequate from providers prospective?*

- If maternal care services payments do not exist in Georgia:

Show again Card 1 to the respondent and ask about the opinion of the respondent in general:

*What levels of payment would be adequate for these services for providers?*

After the responses from this prompt have been exhausted, move on.

- - Do you think that the payments for maternal care were the problem for you to access/use certain services? Is it resulting low utilization form your side? Have you taken any loan from your family, friends or bank to cover health expenditure? Was it that related with maternity?
  - Did you ever have to pay for maternal care unofficially in cash or kind?
    - If yes, what was the reason for it? (e.g. gratitude, ensuring quality, it is widely accepted method).
    - Have you asked to give some amount or gift to the medical staff? if yes,

Was it direct negotiation or hint?

After the responses from this prompt have been exhausted, move on.

*-*How many of you experienced C-section? Was it mostly emergency need or planed? If it was planned C-section why you decided to do so? What is your opinion about C-section? How much have you paid for C-section?

- - How many of you experienced any pregnancy, childbirth or postpartum complication, like preeclampsia, haemorrhage, etc? If any, what was the outcome?
  - How many of you experienced stillbirth, preterm childbirth or infant mortality?

-Was that a barrier to seek for the health service?

- How you will describe the health provider where you received last services? Hypothetically how you will grade it? Was it high or low level services?

**III. GENERATION OF A LIST WITH ASSESSMENT CRITERIA (20 MINUTES)**

**• Brainstorm on relevant assessment criteria**

*Now, we are going to brainstorm. I have the following question for you.*

Show Card 2 to the participants and read the text on it.

| CARD 2 | Imagine that you need to assess the adequacy of the maternal healthcare services that are implemented or considered for implementation in a country.  What assessment criteria would you take into account? please see the list below:   - Safety - Delay of care - Accountability - Confidentiality - Fee for services - Consultation time - Facility outlook | 2015  Georgia |
| --- | --- | --- |

*Please, keep in mind that for us, there are no right or wrong answers. We are interested in your personal opinion. Please, also keep in mind that we aim at a broad range of assessment criteria (incl. economic, social, institutional, geographical, ethical, cultural and demographic).*

This is primarily a brainstorm exercise. Participants can add as many ideas as they occur to them.

Prompt for economic, social, institutional, historical geographical, ethical, cultural and demographic.

After the responses from this prompt have been exhausted, announce the break.

BREAK: 10 MIN.

**IV. GENERAL DISCUSSION ON MATERNAL HEALTHCARE (25 MINUTES)**

- Ask the participants to reflect on the discussion.

*Is there anything we have not discussed that seems relevant to you?*

After the responses from this prompt have been exhausted, move on.

- Ask the participants to reflect on the link between payments for maternal healthcare services and quality of care.

*What do you think about payments for maternal healthcare in the health care sector?*

*Do you think that there is relation between quality and financial access?*

After the responses from this prompt have been exhausted, move on.

- Ask the participants to reflect on the link among health care reforms and quality and accessibility of maternal healthcare

*What do you think, does changes that happened during last period in healthcare influence access to maternal healthcare services?*

*What do you think, about relaibility of the health care reform (universal coverage)?*

*What do you think, does maternal health priority for the government?*

- Make sure that the discussion has been comprehensive.

*Would anyone like to make any final comments?*

After the responses from this prompt have been exhausted, move on.

## **V. CLOSING PART (5 MIN)**

- **Thank the participants**

*This is the end of our focus group discussion. Thank you very much for your input during the discussion. Your opinion is highly valuable for our study.*

- **Explain once again what will happen with the information collected**

*As I explained at the beginning, this group discussion had purely academic purposes. No one outside of this room will have access to these tapes. The information will be analysed as a whole and participants' names will be never used in any analysis of the discussion or in any report.*
